# Supplementary material for: Spectral technique with convergence analysis for solving one and two-dimensional mixed Volterra-Fredholm integral equation
Source: PLoS One. 2023 May 26;18(5):e0283746. doi: 10.1371/journal.pone.0283746 (PMC10218756; doi:10.1371/journal.pone.0283746)
Supplement: Attachment — Submitted filename: Review_report_PONE-D-23-02279.pdf [file pone.0283746.s001.pdf]

Review report of
"Spectral technique with convergence analysis for solving multi-dimensional mixed Volterra-Fredholm integral equation"

A. Z. Amin, A. K. Amin, M. A. Abdelkawy, A. A. Alluhaybi and I. Hashim

1 Summary of the Content

The paper discusses the analysis and the numerical solution of mixed Volterra-Fredholm integral equations (MV-F-IEs). The numerical approach is based on shifted Jacobi-Gauss collocation (SJ-G-C) method. The proposed technique with shifted Jacobi-Gauss (SJ-G) nodes is applied to diminish the MV-F-IEs to a system of algebraic equations that are easily solved. The present algorithm is extended to solve the two-dimensional MV-F-IEs. Convergence analysis for the present method is discussed and confirms the exponential convergence of the spectral algorithm. Various numerical examples are approached to demonstrate the power and accuracy of the technique.

2 Overall Feedback

The work is meaningful and has sufficient novelty, although the same technique seems to be applied to other models by the co-authors. However, the analysis is technical and concrete, and the results are fascinating. The overall structure seems to be logical; definitions are given most of the time. Thus, I suggest the acceptance of the paper for publication. Before that, the authors should consider the following minor comments.

3 Minor comments

1. On page 11, Gronwall → Grönwall.
2. On page 17, the TCM method should be cited correctly.
3. In section 6, please specify the absolute and maximum absolute errors (MAEs) used to report the errors and computational orders.
4. Please, check all the references and capital the following words:
   - volterra → Volterra
   - fredholm → Fredholm
   - hammerstein → Hammerstein
   - chebyshev → Chebyshev
   - legendre → Legendre
   - galerkin → Galerkin
   - jacobi → Jacobi
   - abel's → Abel's
   - adi → ADI
   - crank-nicolson → Crank-Nicolson
   - schrödinger → Shrödinger
5. English language editing/correction needs to be done. For example, on page 2, we notify the following corrections:
   - Line 2: such as theory → include the theory
   - Line 3: in population → population
   - Line 8: challenge → a challenge
   - Line 8: On the light of the foregoing → In light of the preceding
   - Lines 9 and 11: technique have proposed → techniques have been proposed

- Line 12: , see for example → ; for example,
- Line 16: Recently, in [18] the authors → Recently, in [18], the authors
- Line 22: the spectral → spectral
- Line 23: has applied in few scopes such as → have applied in a few scopes, such as
- Line 24: newly → Newly
- Line 28: kind → kinds
- Line 30: to almost satisfying → almost to satisfy
- Line 33: two dimensional → two-dimensional
- Line 34: such equation → a such equation
