# Supplementary material for: Spectral technique with convergence analysis for solving one and two-dimensional mixed Volterra-Fredholm integral equation
Source: PLoS One. 2023 May 26;18(5):e0283746. doi: 10.1371/journal.pone.0283746 (PMC10218756; doi:10.1371/journal.pone.0283746)
Supplement: Attachment — Submitted filename: Responses.pdf [file pone.0283746.s002.pdf]

Dear Prof. Mahmoud A. Zaky
Academic Editor of PLOS ONE

Thank you very much for the referee reports sent some time ago for the above paper. We are submitting the revised version of the paper for publication in "PLOS ONE". We have responded to all the referee helpful comments. It is our hope that the revised version of the paper satisfies you.

I) With respect to the first referee (Reviewer: 1):

1. We carefully checked our manuscript and corrected the errors throughout the paper.
2. The main motivation of the method added in the introductory section.
3. We checked the references list and inserted additional references.
4. We have reviewed and corrected all of the reviewer's required changes.
5. All additions were highlighted in red color.

II) With respect to the second referee (Reviewer: 2):
The main advantages of the present study are:

1. We carefully checked our manuscript and corrected the errors throughout the paper.
2. The main motivation of the method added in the introductory section.
3. We inserted references for the properties mentioned in Section 2.
4. For the two models in 3.1 and 3.2, We inserted suitable references.
5. We compared our work with https://doi.org/10.1007/s40065-019-0243-y.(ex3).
6. We mentioned the platform used to write the codes as well as the specifications of the machine used to debug these codes.
7. We cited only relevant work; also used the cite package.
8. we inserted a Comment on future extensions in this direction in the conclusion section.
9. All additions were highlighted in red color.

II) With respect to the Editor:

1. We carefully checked our manuscript and corrected the errors throughout the paper.
2. The main motivation of the method added in the introductory section.

3. We replaced "multi-dimensional" by "one- and two-dimensional"in the title.
4. We inserted a comparison with proposed high dimensional spectral methods for the same problems in literature.
5. We added references for lemmas in section 5.1.
6. We checked the references list and inserted additional references.
7. The reference list has been updated, and the style has been unified.
8. All additions were highlighted in red color.

Finally, we would like to thank the Editorial Board of "PLOS ONE" for managing the review process for this manuscript.

Thank you very much in anticipation, I remain
Yours Sincerely
M A Abdelkawy
